# Design modification of surgical drill bit for final osteotomy site preparation towards improved bone-implant contact

**DOI:** 10.1016/j.heliyon.2023.e16451

**Published:** 2023-05-26

**Authors:** Pravin Vasudeo Vaidya, Abir Dutta, Suparna Rooj, Rahul Talukdar, Komal Bhombe, Venkata Sundeep Seesala, Zahiruddin Quazi Syed, Tapas Kumar Bandyopadhyay, Santanu Dhara

**Affiliations:** a Advanced Technology Development Centre, IIT Kharagpur, West Bengal, India; b Metallurgical and Material Engineering, IIT Kharagpur, West Bengal, India; c School of Medical Science and Technology, IIT Kharagpur, West Bengal, India; d Sharad Pawar Dental Collage and Hospital, Dutta Meghe Institute of Medical Science, Wardha, Maharashtra, India; e Jawaharlal Nehru Medical College, Dutta Meghe Institute of Medical Science, Wardha, Maharashtra, India

**Keywords:** Drill bit, Necrosis, Frictional heating, Bone implant contact, Osseointegration

## Abstract

Implant stability significantly impacts accelerated osseointegration, leading to faster patient recovery. Both primary and secondary stability necessitates superior bone-implant contact influenced by the surgical tool required to prepare the final osteotomy site. Besides, excessive shearing and frictional forces generate heat causing local tissue necrosis. Hence, surgical procedure necessitates proper irrigation with water to minimize heat generation. Notably, the water irrigation system removes bone chips and osseous coagulums, which may help accelerate osseointegration and improve bone-implant contact. The inferior bone-implant contact and thermal necrosis at the osteotomy site are primarily responsible for poor osseointegration and eventual failure. Therefore, optimizing tool geometry is key to minimizing shear force, heat generation, and necrosis during final osteotomy site preparation.

The present study explores modified drilling tool geometry, especially cutting edge for osteotomy site preparation. The mathematical modeling is used to find out ideal cutting-edge geometry that facilitates drilling under relatively less operational force (0.55–5.24 N) and torque (98.8-154.5 N-mm) with a significant reduction (28.78%–30.87%) in heat generation. Twenty-three conceivable designs were obtained using the mathematical model; however, only three have shown promising results in static structural FEM platforms. These drill bits are designed for the final drilling operation and need to be carried out during the final osteotomy site preparation.

## Abbreviations

ρ: Half-point angle
$\alpha_{0}$: Clearance angle at the periphery of the drill
φ: Chisel Edge angle
2*t*: Lip thickness
$\delta_{0}$: Helix angle at the periphery
θ_n_: Normal Shear angle
δ: Helix angle at any radius
*l*: Length of lip
*d*′: Length of the chisel edge
R: Outer radius of a drill bit
*f*: feed per revolution
*t*_1_: Depth of cut
*V*_*C*_: Cutting velocity
${dF}_{s}$: Elemental shear force
${dF}_{l}$: Elemental Thrust Force
dM: Elemental torque
${dF}_{c}$: Parallel to the direction of cutting velocity
${dF}_{T}$: Force perpendicular to cutting velocity
${dF}_{R}$: Resultant force
${dF^{\prime }}_{c},{dF^{\prime }}_{T},{dF^{\prime }}_{T}$: Force components
$\varphi_{n}$: Normal shear angel
$k_{db}$: Shear flow stress
$\lambda_{n}$: Normal friction angle
$\varphi_{n}$: Normal Shear angle
$\varphi_{d}$: Dynamic Shear angle
$\eta_{n}$: Chip flow angle
$r_{0}$: Chisel edge angle extremity
$\gamma_{d}$: Dynamic rake angle
$\gamma_{f}$: Angle between cutting speed and feed speed
$\gamma_{w}$: Half chisel edge angle
${dF}_{c}$: Elemental Thrust force (chisel edge model)
${dC}_{c}$: Elemental torque (Chisel edge model)

## 1 Introduction

Implants, such as bone plates, hip implants, knee implants, etc., are usually fixed to the bone using screws, whereas dental implants are set directly into the bone. In both cases, screws, and dental implants, the primary implant stability (PIS) is achieved by threads provided on the surface [[1], [2], [3], [4]]. In contrast, secondary implant stability (SIS) is attained when sufficient osseointegration is achieved [1,[4], [5], [6]]. PIS and SIS depend on tissue health and excellent bone-implant contact (BIC) at the interface. Sufficient osseointegration can be achieved using excellent BIC [7]. Moreover, several studies suggest that the implants/screws with circular/parabolic cross-section areas can achieve excellent implant stability at the apical region instead of flat cross-sections [8]. PIS is essential to provide adequate time to attain contact or distance osteogenesis [[9], [10], [11]]; however, micro motions and inferior bone implants may delay the healing process [1].

**Fig. 1:**
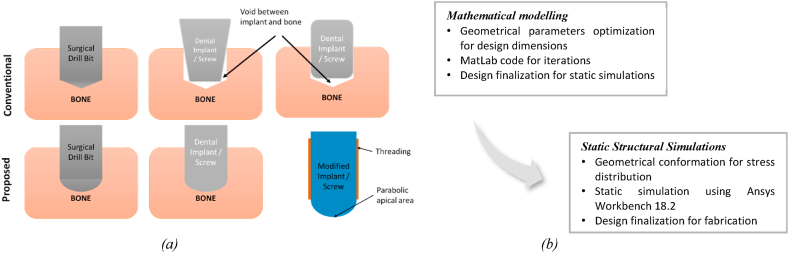
(a) Comparison between Conventional and modified drill bit - implant model; (b) Design strategy for novel drill bit geometry.

Successful implantation necessitates a surgical procedure to prepare an osteotomy site analogous to the implant geometry. The osteotomy site preparation for dental implants and screws requires a drilling operation wherein the native tissues are sheared off from the bone to create a hole [12,13]. However, the commercially available drilling tools have cone angles and straight cutting edges (Fig. 5(a) and (c)) that lead to the formation of the conical cross-section at the apical region [12]. Due to the conical cross-section at the apical region, implants/screws with both flat and parabolic/circular cross-sections show a void space between bone and implant at the apical region (Fig. 1(a)). This particular void affects the BIC, thereby implant stability at the apical region [8,14]. Furthermore, depending on bone mineral density, excessive shearing, and frictional forces during the material removal process generate excessive heat during osteotomy site preparation [13,[15], [16], [17], [18]]. Excessive heat generation is responsible for local tissue necrosis, possibly leading to thermal necrosis [19]. Up to 95% of the energy generated is dissipated in the 5-μm thickness of the contacting bodies [20,21]. The bone tissues/cells of 5-μm thickness are most important for contact/distance osteogenesis [[9], [10], [11]] and osseointegration. Prevention of thermal necrosis in the contact tissue layer at the implant-tissue interface would be a priority towards favoring osseointegration. As a standard surgical practice, the water irrigation system is provided to minimize the heat generation during site preparation [17,[22], [23], [24], [25]]; however, an irrigation system is responsible for removing bone chips and osseous coagulum, which may help to accelerate osseointegration [25,26].

Preparing an osteotomy site analogous to screw/dental implant geometry is crucial to attaining excellent implant stability and a lower implant rejection rate. Hence, it is essential to develop a tool geometry that may create a parabolic cross-section at the close end of the osteotomy site and reduce the effect of thermal necrosis. As a consequence, the proposed study may promote both contact and distance osteogenesis as addresses both issues. Assuming the drill bit is employed for finishing operation during osteotomy site preparation, the mathematical approach is used to design the tool and associated geometrical parameters. The model is then established in MatLab towards optimized geometrical design with minimum force and torque requirement, as shown in Fig. 1(b). Further, the 3D model was designed in CATIA V5 using the optimized geometrical design obtained from Matlab results. The 3D designs/models were further evaluated for validation utilizing Ansys workbench 18.2, keeping the optimized force and torque as boundary conditions.

## 2 Methodology

### 2.1 Optimization of geometrical parameters

**Fig. 2:**
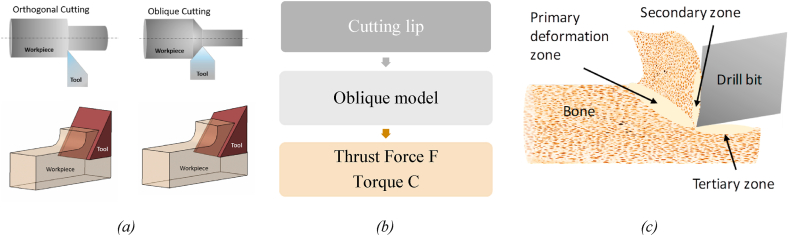
(a) Orthogonal cutting mechanism for chisel edge and Oblique cutting mechanism for cutting lip [30], (b) Mathematical design approach for geometrical parameters optimization [27].

The theoretical design is based on the model explained by Elhachimi et al. [27]. They considered an oblique cutting model for cutting lip and orthogonal cutting models for chisel edges (Fig. 2(a)). The present model is entirely analytical and does not need preliminary experimental results [27]. The mathematical model is based on force and torque continuity; therefore, it is possible to calculate the force and torque as per the tool geometry [27,28]. Hence, the main cutting-edge geometry is modified from straight to parabolic to achieve a round/parabolic apical area at the osteotomy site. The proposed tool design applies to the final osteotomy site preparation before implantation and may be assumed to be used for finishing operations during bone drilling. The proposed drill bit would require a step-drilling/Predrilling/guide hole to prepare the osteotomy site for implantation. Predrilling eliminates the need for a chisel edge while drilling operations and factors such as thrust force, torque, friction, etc., arising from the chisel edge. Therefore, the present model represents the forces and torque owing to the cutting lip of the drill bit (Fig. 2(b)). Although the present study has considered the oblique cutting model for cutting lip, in most studies, it is observed that bone chips formed during drilling operation undergo shear failure that is similar to chip formation during metal machining. Therefore the orthogonal cutting model is considered for heat generation during drilling operations [12,29].

**Fig. 3:**
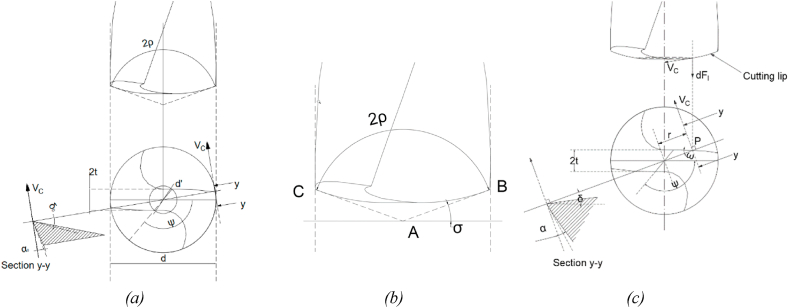
(a) Tool geometry with top and front along with section view showing various angles and (b) side view of drill bit (c) Tool geometry with force and cutting velocity acting at point P.

The geometrical parameters are shown in Fig. 3(a). The present study considers the parabolic profile for cutting-edge geometry; therefore, the parabolic equation can be obtained by considering the point angle formed by the line AB and AC. Line AB and AC are the tangents to the parabola at any radius (R) and at points B and C (Fig. 3(b)). The edge AB and AC make an angle σ with the horizontal axis and can be obtained as,

(1) σ=90−ρ

Let us consider an upward parabolic equation as

(2) $$y=r^{2}$$

where:

r is any point on cutting lip along the x-direction
y is any point along the y-direction.

**Fig. 4:**
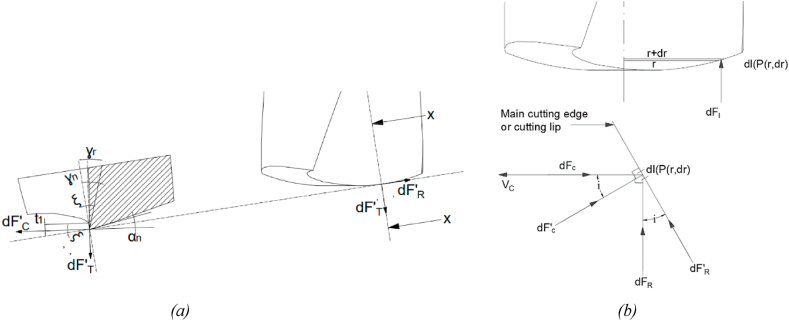
(a) Force acting on the element P at a distance r from the center of the drill bit (b) The elemental forces and associated angles at any point P on cutting edge.

The elemental thrust force dF and torque dC evaluation is done by considering a small element dl over the cutting lip at any point P positioned at a distance ‘r’ from the center of the bit. The cutting tool geometry at point P is such that the cutting speed V_c_ tangent at point P is not perpendicular to dF and dC (Fig. 3(c)). Further, from Fig. 3(c) and Fig. 4(a) the geometrical parameters of the drill bit can be calculated as [27].

(3) $$\omega =\sin^{-1}\left(\frac{t}{r}\right)$$

(4) $$\xi =\tan^{-1}\left(\tan\left(\omega \right)\cos\left(\rho \right)\right)$$

(5) $$\gamma_{r}=\frac{\tan\left(\delta \right)\cos\left(\omega \right)}{\sin\left(\rho \right)-\cos\left(\rho \right)\tan\left(\delta \right)\sin\left(\omega \right)}$$

(6) $$\delta =\frac{d\;\tan\left(\delta_{0}\right)}{2r}$$

Here,

ω is an angle between the line perpendicular to the section line and the cutting lip at any radius r
ξ is an angle between the tangent to the cutting lip and elemental for dF'_c_ at any radius r
$\gamma_{r}$ is an intermediate angle along the cutting lip at any radius r
ρ is a half-point angle,
t is half lip thickness,
r is the radius of a point situated at the cutting edge,
δ is the helix angle at any point.

**Fig. 5:**
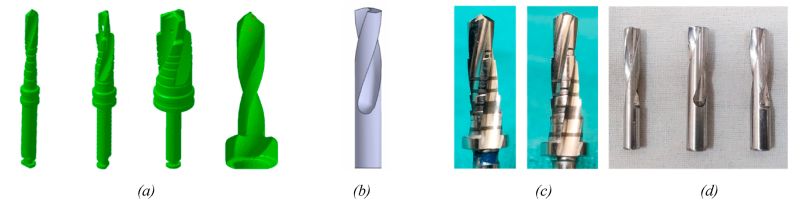
Comparison of surgical drill bits with the fabricated tool used as finishing tool based on the simulation outcome (a) isometric view of the MicroCT scanned commercially available drill bit (b) CAD model of the design as per the mathematical model (c) drill bit for final osteotomy site preparation [45] (d) the fabricated drill bits by 5 axis CNC machining as per the design outcome of the simulation work.

The incremental force and torque are modeled by assuming the cutting geometry is approximately static at any point P along the cutting edge [27,30]. Therefore, the associated inclination and rake angle at point P is given as

(7) $$i=\sin^{-1}\left(\sin\left(\omega \right)\sin\left(\rho \right)\right)$$

(8) $$\gamma_{n}=\gamma_{r}-\xi$$

Further, the total length of the cutting edge can be expressed as,

(9) $$Total\;lengh\;\left(l\right)=Chisel\;edge\;length\;\left(l_{1}\right)+Cutting\;lip\;length\;\left(l_{2}\right)$$

Although the cutting edge and flank combined to form the chisel edge, as per our predrilling hypothesis, we can exclude the chisel edge while predicting thrust force and torque. Therefore,

(10) $$Total\;lengh\;\left(l\right)=Cutting\;lip\;length\;\left(l_{2}\right)=length\;of\;parabola$$

Therefore, from equation (2) and equation (10),

(11) $$l=l_{2}=\int_{pl}^{r}\sqrt{1+{\left(\frac{dy}{dr}\right)}^{2}}$$

Where pl is a half chisel edge length and can be expressed as,

pl=t×tan⁡{90−(180−ψ)}

(12) l=sinh−1(2r)4−sinh−1{2t×tan(ψ−90)}4+r×(r2+14)−t×tan(ψ−90)×{t2×tan(ψ−90)2+14}

The length of differential element *dl* along the cutting edge at point P can be expressed as,

(13) dl=r2+14+r2r2+14+12×4r2+1dr

Further, the depth of cut ‘*t*_*1*_’ at any point P can be expressed from cutting geometry in terms of feed rate as,

(14) $$t_{1}=\frac{f\;\sin\left(\rho \right)\cos\left(\xi \right)}{2}$$

The increments in differential force and torque at point P are modeled utilizing the oblique cutting model established by Oxley and Elhachimi et al. [27,31]. Following Elhachimi et al. model, the proposed mathematical model assumes that the resultant force transmitted by the shear plane and tool/chip interface is in equilibrium. Therefore, the model analyzes stress acting along these shear planes and over the tool/chip interface [27]. The differential element shear force at point P can be expressed as,

(15) $${df}_{s}=\frac{k_{AB}t_{1}dl}{\sin\;\varphi_{n}}$$

where,

*φ*_*n*_ is a normal shear angle
k_AB_ is the shear flow stress of the bone.

From equations (13), (14)), replacing the value of t_1_ and *dl* in the above equation,

(16) dfs=kABfsin(ρ)cos(ξ)[(r2+14)12+r2(r2+14)12+12×(4r2+1)12]2sin∅ndr

Further, the thrust force can be represented in terms of its trigonometric components such that differential force dF_C_ acting parallel to the cutting speed V_c_, the differential force dF_T_ along the direction perpendicular to the cutting speed at point P, differential force dF_R_ in the direction perpendicular to both dF_C_ and dF_T_ [27] (Fig. 4(a) and (b)) [27]*.* The trigonometric component are acting on infinitesimally small elements following force and torque continuity. Hence, from the Elhachimi et al. model [27];

(17) $${dF^{\prime }}_{c}={dF}_{s}\frac{\cos\left(\varphi_{n}-\gamma_{n}\right)}{\cos\left(\theta_{n}\right)}$$

(18) $${dF^{\prime }}_{T}={dF}_{s}\frac{\sin\left(\varphi_{n}-\gamma_{n}\right)}{\cos\left(\theta_{n}\right)}$$

And

(19) $${dF^{\prime }}_{R}=\sqrt{\left({dF^{\prime }}_{C}^{2}+{dF^{\prime }}_{T}^{2}\right)}\;\sin\left(\lambda_{n}\right)\tan\left(\eta_{c}\right)$$

Where dF_C_ is normal to the cutting edge in the plan of cutting edge and the cutting speed. dF’_T_ is normal to the machined surface, and dF’_R_ is normal to dF’_C_ and dF’_T._ The elements of forces dF_C_, dF_T,_ and dF_R_ can be expressed as [27],

(20) $${dF}_{C}={dF^{\prime }}_{C}\;\cos\left(i\right)+{dF^{\prime }}_{R}\;\sin\left(i\right)$$

(21) $${dF}_{T}={dF^{\prime }}_{T}$$

And,

(22) $${dF}_{R}={dF^{\prime }}_{R}\;\cos\left(i\right)-{dF^{\prime }}_{C}\;\sin\left(i\right)$$

Where dF_C_ and dF_R_ are perpendicular to each other in the plane of dF’_C_ and dF’_R_. The angle ‘*i’* is formed between dF’_C_ and dF’_R_. Both dF_T_ and dF’_T_ are alike shown in Fig. 4(a) and (b) [27].

The thrust force element dF_*l*_ and torque dC_*l*_ can be expressed in terms of the elemental force component acting on point P can be expressed as [27];

(23) $${dF}_{l}=\sqrt{{dF}_{T}^{2}+{\left({dF}_{c}\;\cos\left(i\right)+{dF}_{R}\;\sin\left(i\right)\right)}^{2}}\times \sin\left(\lambda_{n}-\gamma_{n}-\xi \right)\sin\left(\rho \right)-\left({dF}_{c}\;\sin\left(i\right)-{dF}_{R}\;\cos\left(i\right)\cos\left(\rho \right)\right)$$

And,

(24) $${dC}_{l}={rdF}_{C}$$

The total thrust force acting on the cutting lip can be evaluated by integrating the elemental thrust force over the length of the parabolic cutting edge

(25) $$F_{l}=n*\int_{pl}^{r}\frac{{dF}_{l}}{dr}dr$$

And,

(26) $$C_{l}=n*\int_{pl}^{r}\frac{{dC}_{l}}{dr}dr$$

Moreover, the frictional coefficient (μ) responsible for frictional heating can be calculated from,

(27) $$\mu =\tan\;\lambda_{n}$$

where,

$\lambda_{n}$ is the normal friction angle acting over the cutting lip

Further, as observed in most studies, bone chip formation follows a similar mechanism as metal chip formations. Therefore, three deformation zones, viz. primary, secondary, and tertiary deformation zones, can also be noticed in bone cutting/drilling operations. Cutting energy involved in shearing bone in the primary zone is primarily responsible for heat generation during drilling operations. The generated heat is decapitated into bone chips, and the drill bit is in the secondary zone. In the tertiary zone, heat generates as a result of friction between the bone and the drill bit [18,29], as shown in Fig. 2(c). The heat generation due to shearing and friction is well explained by Lee J et al. for bone drilling [16]. For calculating the heat generations, authors have directly adopted the method illustrated by Lee J et al. The heat generation due to shearing operation in the primary zone can be expressed as,

(28) $$Q_{shearing}=\frac{N\zeta \tau_{s}t_{c}bVcos\left(\alpha_{n}\right)}{\sin\left(\emptyset_{n}\right)\cos\left(\emptyset_{i}\right)\cos\left(\emptyset_{n}-\alpha_{n}\right)}$$

Heat generation associated with friction between the tool and the bone in the tertiary zone can be expressed as,

(29) $$Q_{friction}=\frac{N\tau_{s}t_{1}bVsin\left(\lambda_{n}\right)\sin\left(\alpha_{n}\right)\cos\left(i\right)}{\left[\sin\left(\varphi_{n}\right)\cos\left(i\right)\cos\left(\theta_{n}+\varphi_{n}\right)\cos\left(\theta_{i}\right)\cos\left(\varphi_{i}\right)+\sin\left(\theta_{i}\right)\sin\left(\varphi_{i}\right)\right]\cos\;\eta_{\lambda }\;\cos\left(\emptyset_{n}-\alpha_{n}\right)}$$

where,

ζ is a constant of proportionality and can be calculated from the model explained by Lee J et al.
N is a number of cutting lips,
τ_s_ is the flow stress here k_AB,_
t_1_ is the depth of cut or uncut chip thickness,
b is the width of cut $b=\frac{\left(R-\frac{d^{\prime }}{2}\right)\;N}{\sin\left(\rho \right)}$,
V is the spindle speed and can be evaluated using $V=\frac{2\pi R\times \left(RPM\right)}{60}$.

$$\theta_{i}=\sin^{-1}\left[\sin\;\lambda_{n}\;\sin\;\eta_{n}\right]$$

$$\theta_{n}=\tan^{-1}\left[\tan\;\lambda_{n}\;\cos\left(\eta_{n}-\alpha_{n}\right)\right]$$

Therefore, adding equations (28), (29)), total heat generation due to drilling operation can be expressed as,

(30) $$Q_{total}=Q_{shear}+Q_{friction}$$

### 2.2 Development of 2D and 3D models

The 3D CAD designs were developed using the geometrical parameters obtained from the mathematical model. These models are then exported into 2D (.acad) and 3D (.iges) formats for detailing and fabrication. Software such as AutoCAD was used to develop the 2D detailing, whereas CATIA V5 was used to create a 3D model (Fig. 5(b)). The details required for the designing and developing of 2D and 3D models are given in Annexure I.

### 2.3 Static Structural Analysis

Following the mathematical model, the static structural module for simulation was adopted to confirm the parameters obtained from the mathematical model. The exported 3D models were fed to static structural simulation with the optimized magnitude and direction of force, and torque was used as boundary conditions. The force and torque were applied on the cutting edge, whereas fixed support was given at the opposite end of the tool. The magnitude of force and torque are expressed in Annexure-1. The tool geometry has complex counters; therefore adaptive mesh type was chosen for the simulation. The 3D models meshed with linear four-noded tetrahedral elements with an aspect ratio of 1 and 3.

Moreover, the non-linear material properties were also chosen for simulation, given that the tool material may not follow the liner elastic curve under the influence of cutting forces and torque [32]. Stainless Steel non-linear material properties for drill bit were selected for the simulation from Ansys standard material directory. The geometrical designs that showed promising results in the static structural simulations were chosen for fabrication.

### 2.4 Mesh convergence study

A mesh convergence study was performed by comparing the results of maximum equivalent stresses in the generated 3D models of drill bits under a compressive load of 2–6 N distributed over the cutting edge. The adaptive mesh type was used in the present study (Fig. 9(d)). A drill bit with a 140° point angle, 15° helix angle, and 0.5 mm radius was selected for mesh convergence studies. Three models, A, B, and C consisting of 158,528, 192,422, and 225,305 elements, were considered for the study. Comparison between model A and model B revealed 8.6% deviations in equivalent stresses, whereas the differences reduced to 3.8% on comparison between model B and C. Hence, model B with 192,422 elements and edge length varying from 0.11 to 0.2 mm was chosen for the study (Fig. 9(c)).

### 2.5 Fabrication

The drill bits have complex, cutting-edge geometry and must confirm the possibility of fabrication. Therefore, the drill bits were fabricated with optimized geometrical parameters. SS 316 material was used as a tool material (Fig. 5(d)). The tools were fabricated using exported 3D design and LMW-JV Kraft 5-axis CNC machine at Keytex Machines in Surat, Gujarat, India. All the tools were compared with the existing surgical drill bits to confirm and extricate tool geometry. Fig. 5(a) and (b) indicate the difference between the micro CT scanned tools and designed tools whereas Fig. 5(c) and (d) indicate the difference between existing and fabricated drill bits.

## 3 Results

The proposed design is based on the theory proposed by Elhachimi et al. for the cutting forces and corresponding torque acting on the straight cutting edges [27]. The straight cutting edges are responsible for the conical cross section at the apical region of the osteotomy site (Fig. 1, Fig. 5). Therefore, to optimize the proposed design's geometrical parameters, it is essential to consider the forces and torque acting on the parabolic cutting edge. The geometrical parameters for existing drill bits have different recommended parameters for bone drilling. Therefore, the proposed design has considered parameters such as feed, point angle, chisel edge thickness, radius, and helix angle as varying parameters. In contrast, chip flow angle, chisel edge angle, and clearance angles are assumed to be constant and assigned a constant value based on the recommended parameter range [19] (Table 1(b)). Moreover, material property such as flow stress is essential in material removal. In the present study, the material is bone; therefore, the flow stress of bone has been considered for further calculations.

**Table 1:** (a) Reported geometrical parameters for commercially available surgical drill bits (b) the constants assumed from the reported values for developing mathematical modeling (c) input parameters considered for obtaining the geometrical parameters using a mathematical model and MatLab iteration [13,18,19,30,31,39,42].

(a) Reported Values	
Chisel edge thickness (t)	0.1 mm
Feed rate	0.1–0.42 mm/rev
Chisel edge angle	125° – 135°
Chip Flow angle	11°
Flow stress for cortical bone K_ab_	51.6 MPa
Rake angle	20° - 30°
Clearance angle	12° - 15°
Point angle	70°- 90^o^, 110°-118^o^, 120°-140°
Drill Diameter (mm)	2, 3, 3.5, 4, 4.3, 5
Normal Shear angle	37° −66°
Normal friction angle	33.58° −36.86°
Friction Coefficient	0.644–0.75
Inclination angle	10.5°
Dynamic Shear angle	Normal shear angle
Dynamic Friction angle	Normal friction angle
Net Force	1.5–9 N, 19.6–117.6 N
(b) Assumed constants	
Chip flow angle	11°
Chisel edge angle	125°
Flow stress K_ab_	51.6 MPa
Clearance angle	18°
(c) Input for iterations	
Feed (f)	0.1–0.5 mm/rev
Radius (r)	0.5–2.5 mm
Helix angle	15° – 35°
Point Angle	70° – 140°

**Fig. 6:**
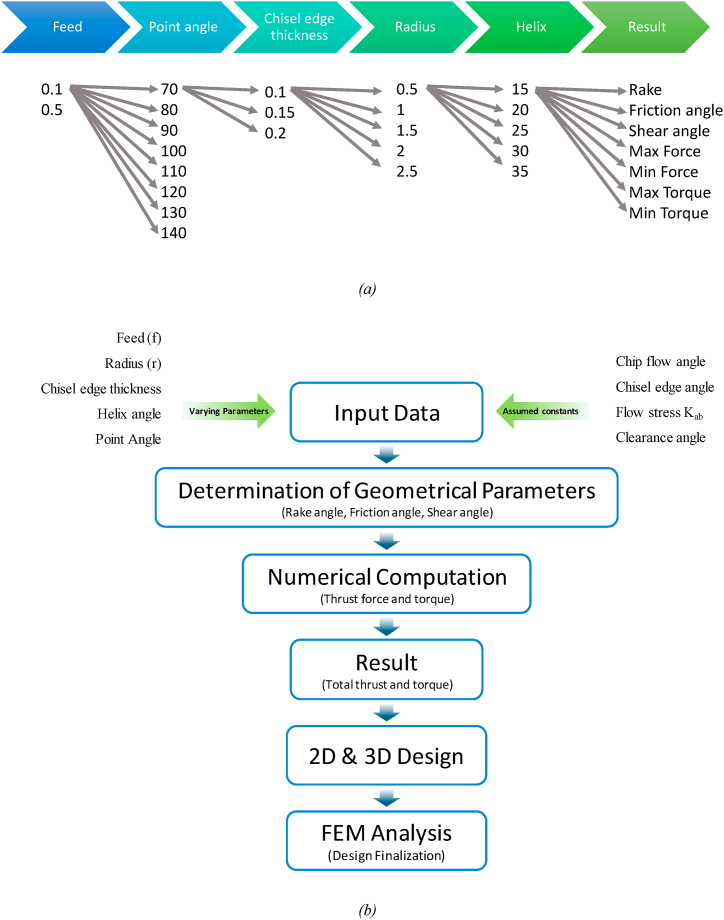
(a) Iteration strategy and parameters considered for geometrical design calculations (b) flow chart for drill bit design.

Additionally, based on the recommended geometrical parameter range, optimization requires repeated iterations, as shown in Fig. 6(a). Optimizing parameters such as thrust, cutting force, the torque required, etc., is highly concerning to avoid necrosis due to excess mechanical and thermal stresses. Notably, the force/torque prediction and approximations are dependent on drill bit geometry. The MatLab code has been developed based on mathematical formulations (equations (1), (2), (3), (4), (5), (6), (7), (8), (9), (10), (11), (12), (13), (14), (15), (16), (17), (18), (19), (20), (21), (22), (23), (24), (25), (26)) to avoid manual calculations and errors. The varying parameters acted as input parameters to the MatLab code and must be fed manually into the code while compiling the same. Further, each geometry has a different equation, leading to subsequent iterations (Fig. 6(a)). Moreover, the model calculated geometrical parameters such as rake angle, shear angle, friction angles, etc. The geometrical parameters with minimum force and torque values were chosen as an optimized design. The corresponding values of heat generation and thermal parameters were also calculated (equations (27), (28), (29), (30))) for the optimized design. These parameters are essential for developing 2D and 3D models for FEM analysis and fabrication. Further, the mesh convergence study has also been performed on the developed 3D models. The flow chart for design finalization is as shown in Fig. 6(b).

### 3.1 MatLab results

The MatLab code was developed based on the mathematical model (equations (1), (2), (3), (4), (5), (6), (7), (8), (9), (10), (11), (12), (13), (14), (15), (16), (17), (18), (19), (20), (21), (22), (23), (24), (25), (26)) (Annexure-V). The inputs and constants (Table 1(b) and (c)) were set forth to the code, and the iterations were performed per the iteration strategy (Fig. 6(a)). Geometrical designs of drill bits have been selected and evaluated based on drill thrust force magnitude and directions acting on the cutting edge, comparable forces, and torque acting on the proposed models. Directions considered in this analysis are upward positive for thrust force and counterclockwise positive for torque. Moreover, the code was developed assuming the Chip flow angle, clearance angle, and chisel edge angle as 11°, 18°, and 125°, respectively, (Table 1(b)). Also, the bone flow stress was assumed to be 51.6 N/mm (Table 1(a)). Notably, geometrical parameters such as a rake, shear, and friction angles were also considered while selecting the optimized drill bit (Fig. 3, Fig. 4). All these geometrical parameters were used to develop the 3D model using CAD. Twenty-three promising design combinations were finalized based on selection criteria as shown in Fig. 7(a)–(d), and Fig. 7(e)), (Annexure-III). With the proposed model, the force required is in the range of 0.13–10.3 N, whereas the torque was 6.34–155.8 N-mm. The obtained force and torque are less than the reported values ranging from 1.5 N to 117 N. Further, the rake angle and friction angle found were to be 12.53°–21.9° and 22.75°–25.44°, respectively, whereas the reported rake angle for bone drilling ranges from 20° to 30° and friction angle falls in the range of 33°–36.83° [19]. Moreover, the frictional coefficient (μ) responsible for frictional heating falls in the range of 0.421–0.445. The shear and friction forces acting in the primary and tertiary zone, respectively, are responsible for heat generation during bone drilling [14]. The combined effect of shear and friction heating was found to be 28.78%–30.87% (Fig. 8).

**Fig. 7:**
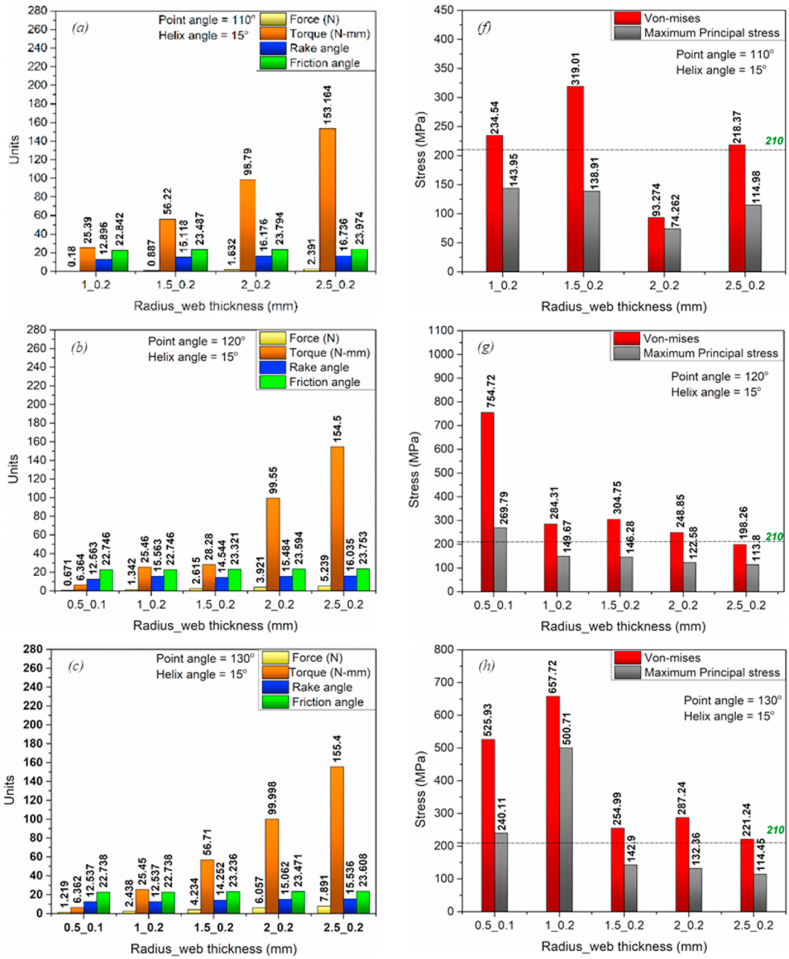
Optimized parameters including force, torque, rake angle, and friction angle (a) Point angle 110° and helix angle 15° (b) Point angle 120° and helix angle 20° (c) Point angle 130° and helix angle 15° (d) Point angle 140° and helix angle 15° (f) Point angle 110° and helix angle 15° (g) Point angle 120° and helix angle 20° (h) Point angle 130° and helix angle 15° (i) Point angle 140° and helix angle 15°.

**Fig. 7:**
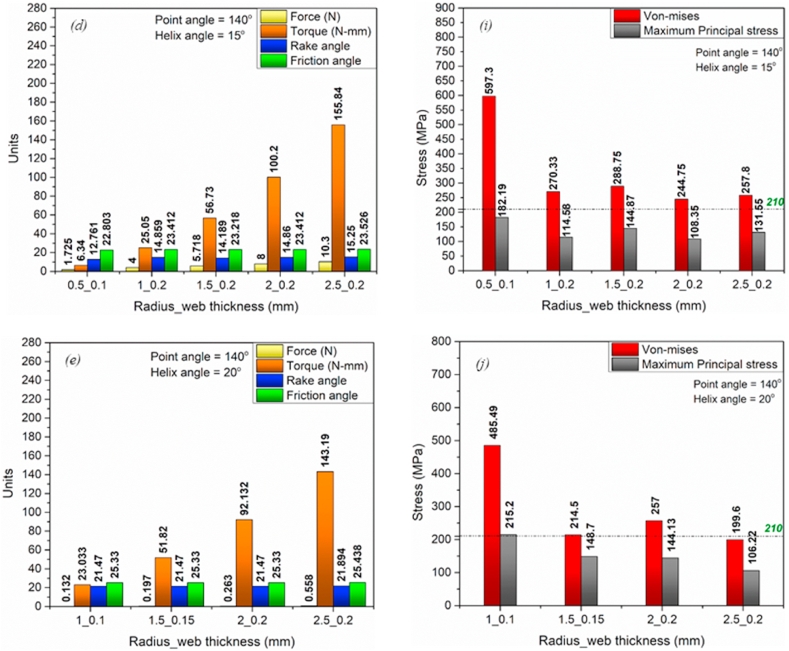
Optimized parameters including force, torque, rake angle, and friction angle (e) Point angle 140° and helix angle 20°. Von mises stress and Maximum principle stress simulated using Static structural FEM simulations for point angles 110°-140° utilizing ANSYS Workbench 18.2 (j) Point angle 140° and helix angle 20°.

**Fig. 8:**
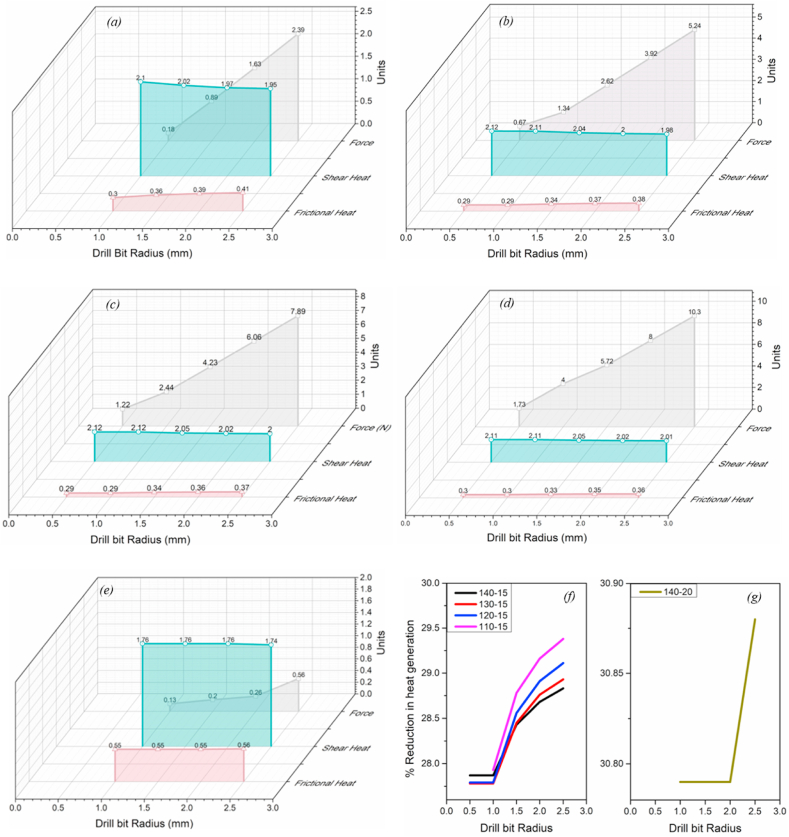
Fractional variation in shear and frictional heat due to the thrust force applied by drill bit during bone drilling as obtained from mathematical model (a) 110° _15° (b) 120° _15° (c) 130° _15° (d) 140°_15° (e) 140°_20°. The percent reduction in heat generated during the drilling operation was evaluated by comparing the existing drill bit with the modified drill bit (f) heat reduction in drill bits with respect to existing drill bits 110° _15°, 120° _15°, 130° _15°, 140°_15°, (g) 140°_20°.

**Fig. 9:**
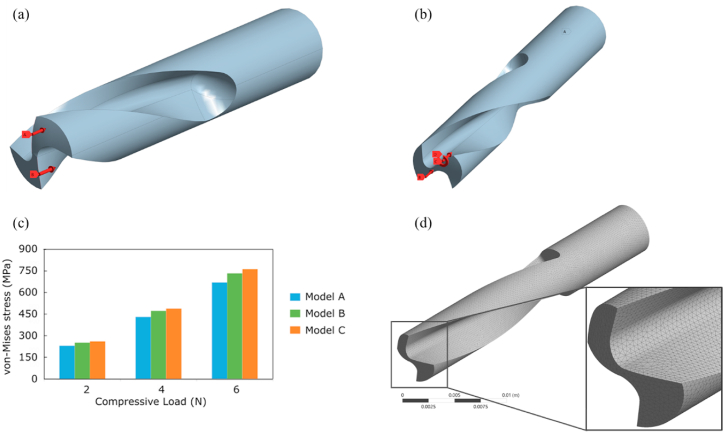
The evaluated Force and torque values were used as boundary conditions for the static structural FEM using Ansys workbench 18.2 (a) thrust force acting at the cutting lip (b) combined torque and forces on cutting lip (c) mesh convergence studies have been performed on the drill bit with 140° point angle and 15° helix angle and 0.5 mm radius. Three models A, B, and C consisting of 158,528, 192,422, and 225,305 elements were considered for the study. (d) Proximity and curvature meshing were used for meshing the drill bit.

### 3.2 Static structural FEM analysis

The mathematical model is best suitable for the prediction of cutting forces and torque acting on the drill bit without the requirement of preliminary experimental results. However, in actual practice, the model does not explain the drill bit as a whole. Therefore, it is essential to validate the results of the mathematical model in virtual 3D geometry. To evaluate the effect of force and torque acting on the cutting edge as well as on the complete drill bit, the FEM technique was adopted. FEM simulations were executed by considering SS Non-linear material [32] with the loading conditions shown in Fig. 9(a), (b), and Table 2. Adaptive mesh type was adopted for simulation (Fig. 9(d)). Since the mathematical model is assumed to be in the statistical condition, performing FEM analysis with similar parameters and conditions is essential. The static structural model was used to simulate all twenty-three designs, and it was found that only three designs out of twenty-three are within the stress limits (Fig. 7(f)–(i), and Figs. 7(j)), and Fig. 8) (Annexure III, IV). The designs were finalized considering the maximum principle stress theory as well as the von-Mises stress theory. Based on our results, it is found that most of the designs are safe as per the maximum-principle stress theory; however, very few designs were found to be safe under von-Mises theory. These results were also expected while finalizing the designs as per the failure theory.

**Table 2:** Drill bit Material and native bone properties (Stainless steel non-linear) for static structural FEM simulation utilizing Ansys Workbench 18.2 [[13], [16], [19], [30], [31], [39], [42]].

Material	Stainless steel NL	Bone
Density	7750 kg/m^3^	1900
Specific Heat	480 J/kg-C	1260
Young's Modulus	193 GPa	17 GPa
Poisons Ratio	0.31	0.3
Bulk Modulus	169.3 GPa	14.167 GPa
Shear Modulus	73.664 GPa	6.538 GPa
Yield Strength	210 MPa	110 MPa

## 4 Discussion

The drill tool geometry is responsible for a conical cross section at the close end of the osteotomy site due to the conical point formed by the straight cutting edge and the flanks (Fig. 5(c)). Pawan S. et al. reported that the close contact between the implant and bone bed improves osseointegration, thereby, secondary implant stability (SIS). Furthermore, a round apical bone-implant contact influences implant stability or SIS, as reported by Fanali et al. and Gehrke et al. [8,14]. A void can be observed between the implant apical area and a close end of the osteotomy site leading to lower BIC due to conical cross-section (Fig. 1(a)). Therefore, preparing an implant/screw analogous osteotomy site is essential to achieve excellent SIS. This approach may result in excellent BIC at the osteotomy site close end and help minimize micromotions, thereby improving secondary implant stability (SIS). To improve BIC and SIS, the drill bit geometry, especially the cutting edge, has been modified from straight to parabolic. In doing so, the conical cross-section is transformed into a parabolic cross-section at the close end of the osteotomy site.

Further, as observed in most studies, bone chip formation follows a similar mechanism as metal chip formations. Therefore, three deformation zones, viz. primary, secondary, and tertiary deformation zones, can also be noticed in bone cutting/drilling operations. Cutting energy involved in shearing bone in the primary zone is primarily responsible for heat generation during drilling operations. The generated heat is decapitated into bone chips, and the drill bit is in the secondary zone. In the tertiary zone, heat causes due to friction between the bone and the drill bit [18,29], as shown in Fig. 2(c). Heat generation during drilling operations is reportedly responsible for local tissue apoptosis or necrosis [19,[33], [34], [35]]. However, heat generation and necrosis can be reduced up to a certain limit by providing irrigation while drilling [15,17,18,[22], [23], [24], [25],^36^,^37^]. Although irrigation prevents thermal necrosis, it removes osseous coagulum and osteotomy autologous bone debris [25,26]. Retention of osteogenic matter during drilling becomes more important for patients with age or trauma-related complaints.

The mathematical model has been developed to attain the bone-implant close fit and lower thermal necrosis. The proposed model reports modified geometrical parameters for the drill bit with a parabolic cutting edge. In addition, it is designed for final osteotomy site preparation prior to implant/screw placement. Several iterations have been performed on the developed mathematical model considering feed, point angle, chisel edge thickness, helix angle, and drill radius resulting in possible geometrical parameters such as rake angle, friction angle, maximum thrust force, and maximum torque (Fig. 7(a)–(d), and Fig. 7(e)).

The rake angle influences the cutting forces acting on the drill bit and directly correlates with the same. Moreover, an increase in thrust force and rake angle can be witnessed with an increase in drill bit radius (Fig. 7(a)–(d), and Fig. 7(e)). Saha et al. pointed out that substantial point and helix angles are responsible for positive rake angles along the cutting lip, which help increase drill bit effectiveness [35]. Furthermore, a decrease in principal cutting forces was observed by Jacob et al. irrespective of bone osteon arrangement with a positive rake angle [29]. The rake angle, maximum force, and torque are in the range of 12.53°–21.9°, 0.13–10.3 N, and 6.34–155.84 N-mm, respectively. In comparison, the previous studies reported that the recommended rake angle and corresponding force are in the range of 20°–30° and 1.5–117.6 N, respectively [19](Table 1(a)). The lower magnitude of cutting forces is evident from the positive rake angle over the cutting lip, as obtained from the mathematical model.

Furthermore, the magnitude of force indicates that the torque required for drilling also falls in the lower range. The energy needed for drilling is affected by the rake angle, which is responsible for reducing specific drilling energy, improving chip flow, and promoting cutting operation [19,35,38]. This enables the use of low-torque drilling machines compared to the existing ones. Besides, lightweight and low torque drilling machines will significantly reduce the human efforts required for handling machines and increase the surgeon’s efficiency.

The heat generation in the cutting process is primarily caused due to shearing of native tissues and friction between the tool and bone [39,40]. The temperature generation above the threshold (45 °C–55 °C) leads to necrosis [19,35]. Moreover, the specific cutting energy is primarily responsible for temperature generation while drilling. In addition, specific cutting energy depends on the bone’s dynamic shear strength. The optimum geometrical parameters, such as shear and rake angles, reduce specific cutting energy [35] that may otherwise lead to thermal necrosis [41].

Further, the shear angle was also found to be in the range of 29.3°–31.05° which is significantly lower than the recommended range of 37°–60° for bone drilling [31,42,43]. However, the shear angle is proportional to the rake angle, which signifies that the lower the magnitude of the rake angle lower is the shear angle. Furthermore, the heat generation due to the shearing at the primary deformation zone is directly related to the cosine of the rake angle, indicating higher the rake angle, the lower the heat generation will be. Whereas, in the case of friction heat generation, a product of the sine of the rake and the sine of the friction angle is directly related to frictional heat generation, indicating that the rake and friction angle should be minimum. This also signifies that the lower the rake and friction angle, the less frictional heat generation is. Notably, the higher rake angle is required for lesser heat generation due to shearing, while on the contrary the lower rake angle is necessary for less heat generation due to frictional heating. Therefore optimized rake angle with a minimum friction angle is required to avoid thermal necrosis [18]. The friction angle at the secondary shear zone was found to be in the range of 22.75°–25.44°.

Additionally, the friction coefficient was calculated to be in the range of 0.421–0.445 and is considered constant throughout the cutting process. However, in several studies, the friction coefficient was assumed to be 0.644–0.75 [13,18], higher than the proposed model. The effect of friction heat generation associated with the friction coefficient and friction forces, and shear heating associated with a rake and shear angle [18,30,31,43] might be 28.78%–30.87% less than the existing drill bits (Fig. 8(f) and (g)). The combined effect of rake angle, shear angle, and friction angle on the force, torque, and heat generation can be observed to be significantly lower as compared to the reported values for bone drilling (Fig. 8(a)–(e)) and Table 2).

The heat generated due to friction and shearing forces is carried out by bone debris, blood, osseous coagulum, and water [19]. The excessive heat may also cause apoptosis to the bone chips and osseous coagulum, which may help in SIS and bone regeneration. The clogging stops heat transfer between bone and tool, leading to necrosis at BIC [35]. A higher helix angle is usually preferred for faster chip removal and avoiding chip clogging. Besides, the helix angle and point angle greatly influence the rake angle, indicating the dependence of specific cutting efficiency on the helix and point angle [35]. Experimental findings of Jacob et al. suggest that the 110° point angle produces less thrust and torque [29]. Notably, the current model has shown possible designs for 110°, 120°, 130° and 140° of point angle. However, a 90° point angle is ideal for surgical drills and 130°–140° for cortical bone drilling, whereas drills bits with a 120°–140° point angle are optimized for the thrust and torque required for drilling [19,29,35]. The helix angle of 13°–35° is preferred for bone drilling [19,35]. However, a 28°-helix angle requires less torque and generates lower heat energy [19]. The mathematical model shows that most 15°–20° helix angle designs suit the proposed drill bit design. It can be observed from the mathematical model that the required torque and thrust for drilling with a 140° point angle decreased with an increase in the helix angle (as shown in Fig. 7(d) and 7(e) and Annexure-II). Similar observations were also made out by Saha et al. Notably, the proposed model also shows a lower frictional coefficient, lower thrust, and torque requirements, which might lead to lower heat generation. Furthermore, the proposed model has a lower helix angle that may retain osseous coagulum and bone debris at the osteotomy site, promoting faster osseointegration [25,26]. These additional advantages could help in avoiding irrigation systems during surgery. So far, few of the experimental models have shown optimized drilling torque and axial drilling force. However, most experimental investigations suggest high speed with larger force or greater feed rate for minimum temperature generation in bone drilling [19,35].

**Fig. 10:**
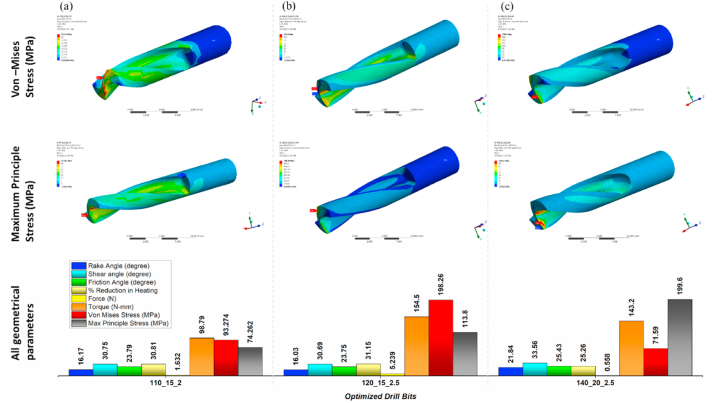
Geometrical parameters of optimized drill bit obtained from the mathematical model and validated using static structural FEM analysis (a) 110°-15°-2 (b) 120°-15°-2.5 (c) 140°-20°-2.5 (Point angle_ Helix angle_ Drill radius).

The mathematical model considers only a static force system, and performing design validation studies under similar conditions are essential. Therefore, similar to the mathematical model, the magnitude and direction of force and torque were considered as boundary conditions for the FEM Analysis (Fig. 9(a) and (b)). In contrast, the material considered in the simulation is stainless steel non-linear (Table 2). Moreover, mesh convergence studies were also performed on the designs. Based on mesh convergence studies, the best mesh size for each design was selected to perform FEM analysis. Any accepted design must be within the material's yield limit. von Mises stresses are obtained from the stress exerted on the object under investigation (herein, cutting edge and whole drill bit). In contrast, the maximum principle stresses are the consequence of uniaxial loading acting around the loading site (herein cutting edge). Therefore it is essential to consider both theories for selecting the tool design. The designs were finalized considering the maximum principle stress theory as well as von –Mises stress theory. In the proposed study, most of the designs are found to be safe in maximum principle stress theory, indicating the drill bit, mainly the cutting edge, is safe to operate and may not get blunt easily. However, few of the designs show safe under the von – Mises theory, indicating that the design is safe to operate under the loading conditions [44]. Comparing maximum principle stress and von Mises stress values in Annexure-III and Fig. 7(f)–(i) and Fig. 7(j)), only three out of twenty-three designs are found to be in the yield limit of the drill bit material as shown in Fig. 10(a) 110_15_2, (b) 150_20_2.5, and (c) 140_20_2.5. The deformation in the drill bits was evaluated in the 10^−5^ m range and can be neglected in the present context.

The study, however, has certain limitations. The mathematical model is limited to 2D geometry, particularly cutting-edge profiles. However, the mathematical model does not explain the combined effect of force and torque acting on the whole drill bit. Therefore, based on results obtained from mathematical modeling, static structural FEM analysis has been performed on the proposed drill design. Nevertheless, quasistatic and dynamic simulations may be explored in the future.

## 5 Conclusion

The mathematical model and static structural FEM analysis were implemented to finalize the drill bit design. Twenty-three drill bit designs have shown promising results in the mathematical model. However, the static structural FEM analysis shows that three designs were found to be safe under similar loading conditions. Moreover, heat generation during the drilling process was also calculated for all twenty-three designs. It was found that the point angle plays an important role in heat generation. An increase in point angle reduces the total heat generation during the drilling operation. Thus, higher point angle drill bits are desirable for reducing thermal necrosis. Notably, all three drill bits fall in the range of the frequently used implant/screw diameters. Therefore, these bits could be implemented in the existing surgical procedures.

## Ethical approval

Not required.

## Author contribution statement

Pravin Vasudeo Vaidya: Conceived and designed the experiments; Performed the experiments; Analyzed and interpreted the data; Wrote the paper.

Abir Dutta: Conceived and designed the experiments; Analyzed and interpreted the data.

Suparna Rooj: Performed the experiments; Analyzed and interpreted the data.

Rahul Talukdar: Analyzed and interpreted the data.

Komal Bhombe: Conceived and designed the experiments.

Venkata Sundeep Seesala: Conceived and designed the experiments.

Zahiruddin Quazi Syed: Conceived and designed the experiments; Contributed reagents, materials, analysis tools or data.

Tapas Kumar Bandyopadhyay, S Dhara: Conceived and designed the experiments; Contributed reagents, materials, analysis tools or data; Wrote the paper.

## Funding statement

The authors have received funding in the form of fellowship from Ministry of Education (MoE). This study also received partial funding from New Generation Innovation and Entreprenurship Development Centre, sponsored by Depertment of Science and Technology- Govt of India with the implimenting institute as Datta Meghe Institue of Higher Education and Research, Wardha, Maharashtra.

## Data availability statement

Data included in article/supplementary material/referenced in article.

## Declaration of competing interest

The authors declare that they have no known competing financial interests or personal relationships that could have appeared to influence the work reported in this paper
