# Supplementary material for: Design modification of surgical drill bit for final osteotomy site preparation towards improved bone-implant contact
Source: Heliyon. 2023 May 26;9(6):e16451. doi: 10.1016/j.heliyon.2023.e16451 (PMC10245014; doi:10.1016/j.heliyon.2023.e16451)
Supplement: Multimedia component 1 [file mmc1.docx]

**SUPPLEMENTARY**

Annexure-I

Half-point angle gives the slope of a line formed at the tip of the drill bit, which is tangent to the parabola at point (r, y). The condition for a line of slope ‘m’ tangent to the parabola at any point(r, y) is,

$r=2am and$

$y=am^{2}$

The slope of line AB or AC (m) is given as

$m=tan(\sigma)$

Therefore, from equations 3 and 4, foci can be expressed as

$a=\frac{r}{2tan(\sigma)}$

| *Calculated values for the equation of parabolic cutting lip for drill bit radius of 0.5 to 2.5 mm and point angle of 70^o^ – 140^o^* | | | | | | | | | | |
| --- | --- | --- | --- | --- | --- | --- | --- | --- | --- | --- |
| **Point angle** | **140** | | **130** | | **120** | | **110** | | **100** | |
| Radius | a | $\frac{tan(\sigma)}{8}$ | a | $\frac{tan(\sigma)}{8}$ | a | $\frac{tan(\sigma)}{8}$ | a | $\frac{tan(\sigma)}{8}$ | a | $\frac{tan(\sigma)}{8}$ |
| 0.5 | 0.685587431 | 0.364650792 | 0.535077196 | 0.467222303 | 0.432112882 | 0.578552528 | 0.356239217 | 0.701775627 | 0.297212367 | 0.841149387 |
| 1 | 1.371174863 | 0.182325396 | 1.070154393 | 0.233611151 | 0.864225764 | 0.289276264 | 0.712478434 | 0.350887814 | 0.594424733 | 0.420574693 |
| 1.5 | 2.056762294 | 0.121550264 | 1.605231589 | 0.155740768 | 1.296338646 | 0.192850843 | 1.068717651 | 0.233925209 | 0.8916371 | 0.280383129 |
| 2 | 2.742349726 | 0.091162698 | 2.140308786 | 0.116805576 | 1.728451527 | 0.144638132 | 1.424956868 | 0.175443907 | 1.188849467 | 0.210287347 |
| 2.5 | 3.427937157 | 0.072930158 | 2.675385982 | 0.093444461 | 2.160564409 | 0.115710506 | 1.781196085 | 0.140355125 | 1.486061834 | 0.168229877 |

| **Point angle** | **90** | | **80** | | **70** | |
| --- | --- | --- | --- | --- | --- | --- |
| Radius | a | $\frac{tan(\sigma)}{8}$ | a | $\frac{tan(\sigma)}{8}$ | a | $\frac{tan(\sigma)}{8}$ |
| 0.5 | 0.249324994 | 1.002707335 | 0.20913581 | 1.195395468 | 0.174437014 | 1.433182064 |
| 1 | 0.498649988 | 0.501353667 | 0.418271621 | 0.597697734 | 0.348874028 | 0.716591032 |
| 1.5 | 0.747974981 | 0.334235778 | 0.627407431 | 0.398465156 | 0.523311042 | 0.477727355 |
| 2 | 0.997299975 | 0.250676834 | 0.836543241 | 0.298848867 | 0.697748057 | 0.358295516 |
| 2.5 | 1.246624969 | 0.200541467 | 1.045679052 | 0.239079094 | 0.872185071 | 0.286636413 |

Annexure-II

| *Optimized design combinations obtained after Matlab coding* | | |
| --- | --- | --- |
| Sr. No | Point angle | Nos of designs |
| 1 | 140 | 9 |
| 2 | 130 | 5 |
| 3 | 120 | 5 |
| 4 | 110 | 4 |
| **Total** | | 23 |

Annexure-III
















| Annexure-IV | | | | | | | | | | | | | |
| --- | --- | --- | --- | --- | --- | --- | --- | --- | --- | --- | --- | --- | --- |
| *Static structural simulation of design combinations obtained using mathematical model utilizing Matlab platform.* | | | | | | | | | | | | | |
| Input Parameters | | | | Matlab Results | | | | Static structural simulation | | | | Image | |
| Point angle  (^o^) | Radius  (mm) | Helix angle  (^o^) | Half web Thickness  (mm) | Rake angle  (^o^) | Friction angle  (^o^) | Max Force  (N) | Max Torque  (N-mm) | Number of meshing Nodes | Nos. of elements | Von mises stress  (MPa) | Maximum Principle stress  (MPa) | Von mises stress | Maximum Principle stress |
| 140 | 0.5 | 15 | 0.1 | 12.761 | 22.803 | 1.725 | 6.34 | 285723 | 188268 | 597.3 | 182.19 | 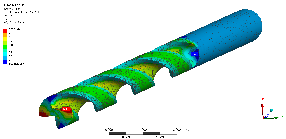 | 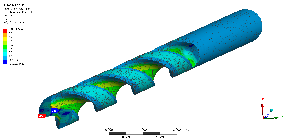 |
|  | 1 | 15 | 0.2 | 14.859 | 23.412 | 4.00 | 25.05 | 116780 | 76979 | 270.33 | 144.58 | 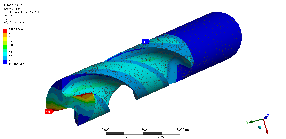 | 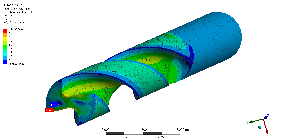 |
|  | 1.5 | 15 | 0.2 | 14.189 | 23.218 | 5.718 | 56.73 | 186278 | 122399 | 288.75 | 144.87 | 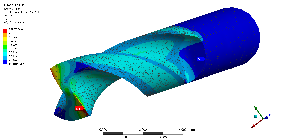 | 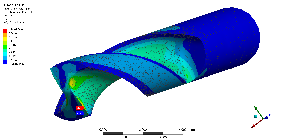 |
|  | 2 | 15 | 0.2 | 14.86 | 23.412 | 8.00 | 100.2 | 139638 | 93668 | 244.75 | 108.35 | 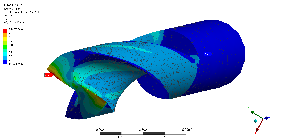 | 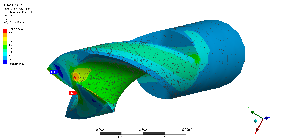 |
|  | 2.5 | 15 | 0.2 | 15.25 | 23.526 | 10.30 | 155.84 | 175198 | 116529 | 257.8 | 131.55 | 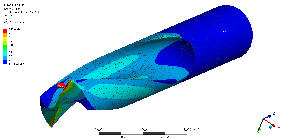 | 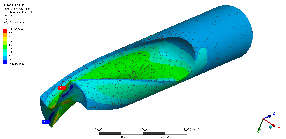 |
|  | 1 | 20 | 0.1 | 21.47 | 25.33 | 0.132 | 23.033 | 220761 | 148350 | 485.49 | 215.2 | 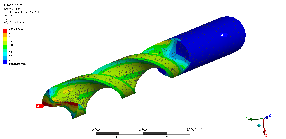 | 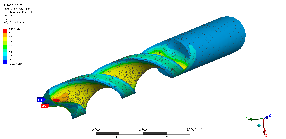 |
|  | 1.5 | 20 | 0.15 | 21.47 | 25.33 | 0.197 | 51.82 | 280600 | 184629 | 241.5 | 148.66 | 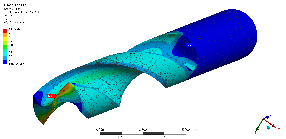 | 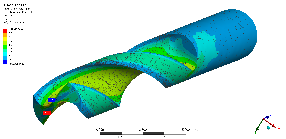 |
|  | 2 | 20 | 0.2 | 21.47 | 25.33 | 0.263 | 92.132 | 140710 | 94019 | 257 | 144.13 | 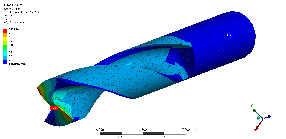 | 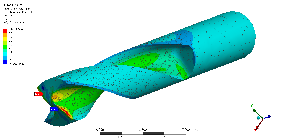 |
|  | 2.5 | 20 | 0.2 | 21.894 | 25.438 | 0.558 | 143.19 | 112993 | 75720 | 199.6 | 106.22 | 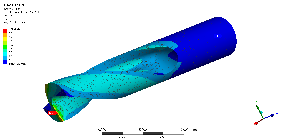 | 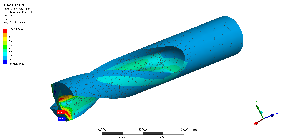 |
| 130 | 0.5 | 15 | 0.1 | 12.537 | 22.738 | 1.219 | 6.362 | 325594 | 218788 | 525.93 | 240.11 | 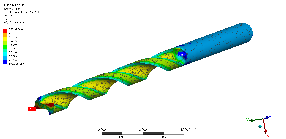 | 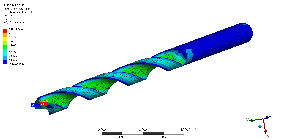 |
|  | 1 | 15 | 0.2 | 12.537 | 22.738 | 2.438 | 25.45 | 111219 | 73860 | 657.72 | 500.71 | 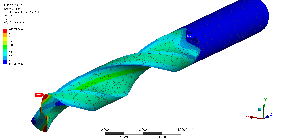 | 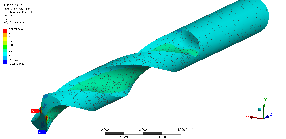 |
|  | 1.5 | 15 | 0.2 | 14.252 | 23.236 | 4.234 | 56.71 | 292420 | 198565 | 254.99 | 142.9 | 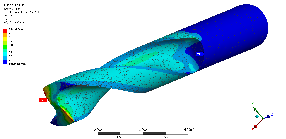 | 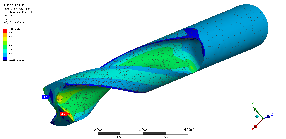 |
|  | 2 | 15 | 0.2 | 15.062 | 23.471 | 6.057 | 99.998 | 112129 | 75522 | 287.24 | 132.36 | 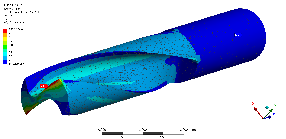 | 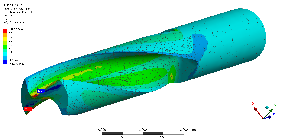 |
|  | 2.5 | 15 | 0.2 | 15.536 | 23.608 | 7.891 | 155.4 | 102088 | 68759 | 225.06 | 105.14 | 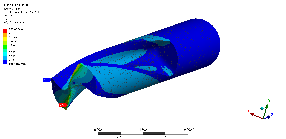 | 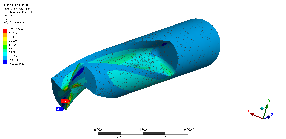 |
| 120 | 0.5 | 15 | 0.1 | 12.563 | 22.746 | 0.671 | 6.364 | 256923 | 176199 | 754.72 | 269.79 | 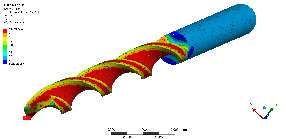 | 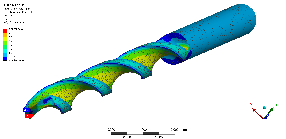 |
|  | 1 | 15 | 0.2 | 15.563 | 22.746 | 1.342 | 25.46 | 97805 | 64835 | 284.31 | 149.67 | 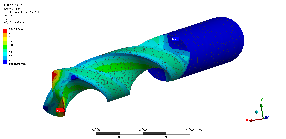 | 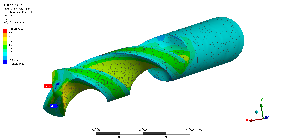 |
|  | 1.5 | 15 | 0.2 | 14.544 | 23.321 | 2.615 | 28.28 | 265002 | 179946 | 304.75 | 146.28 | 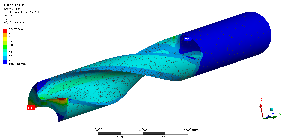 | 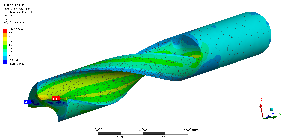 |
|  | 2 | 15 | 0.2 | 15.484 | 23.594 | 3.921 | 99.55 | 116191 | 78375 | 248.85 | 122.58 | 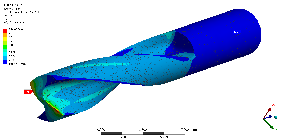 | 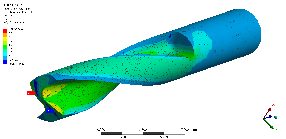 |
|  | 2.5 | 15 | 0.2 | 16.035 | 23.753 | 5.239 | 154.5 | 102063 | 68775 | 198.26 | 113.8 | 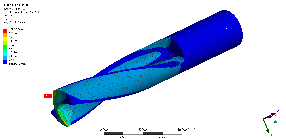 | 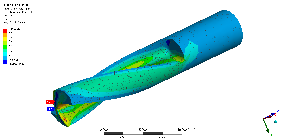 |
| 110 | 1 | 15 | 0.2 | 12.896 | 22.842 | 0.180 | 25.39 | 160908 | 107189 | 234.54 | 143.95 | 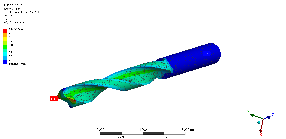 | 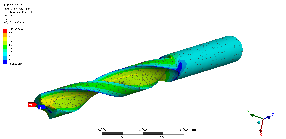 |
|  | 1.5 | 15 | 0.2 | 15.118 | 23.487 | 0.887 | 56.22 | 310255 | 211022 | 319.01 | 138.91 | 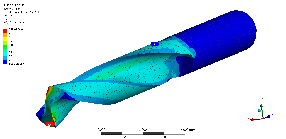 | 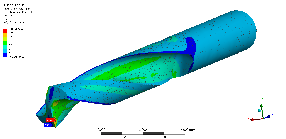 |
|  | 2 | 15 | 0.2 | 16.176 | 23.794 | 1.632 | 98.79 | 107552 | 72401 | 93.274 | 74.262 | 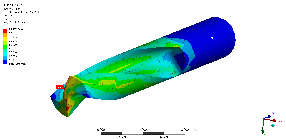 | 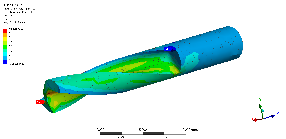 |
|  | 2.5 | 15 | 0.2 | 16.736 | 23.974 | 2.391 | 153.16 | 113894 | 76690 | 218.4 | 114.98 | 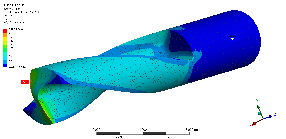 | 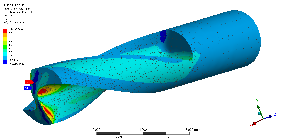 |

Annexure-V

| *Optimized drill bit parameters after mathematical and static structural FEM analysis* | | | | | | | | |
| --- | --- | --- | --- | --- | --- | --- | --- | --- |
| Drill bit  (Point_ helix_ dia) | Rake angle  (^o^) | Shear angle  (^o^) | Friction angle  (^o^) | %Reduction in frictional heating | Maximum force  (N) | Maximum Torque  (N-mm) | Von Mises stress  (MPa) | Maximum Principal stress (MPa) |
| 110_15_4 | 16.17 | 30.75 | 23.79 | 30.81 | 1.632 | 98.79 | 93.274 | 74.262 |
| 120_15_5 | 16.03 | 30.69 | 23.75 | 31.15 | 5.239 | 154.5 | 198.26 | 113.8 |
| 140_20_5 | 21.84 | 33.56 | 25.43 | 25.26 | 0.558 | 143.2 | 71.59 | 199.6 |

Annexure-VI

clc

clear

syms r pl

All_Parameters = [];

Matrix=[];

excelsheet_name = 'Output.xlsx';

% column_names={'Depth','Parameter_A'};

%column_names={'f','t','RR','rho','pl','d0','G','LN','Pn','Tn','X','Fl','Cl','Ft','Ct','d0_2','G_2','LN_2','Pn_2','Tn_2','X_2','Fl_2','Cl_2','Ft_2','Ct_2'};

%for fe = 0.1:0.1:0.5 % feed rate

fe = 0.1;

RR = 0.5 % radius of drill bit 0.5-2.5 mm

rho = (70*3.147)/180 % Hallf Point angle 70°-140°

(# varring parameter)

y = 0.364007*r^2; % equation for parabolla

for f = 0.1:0.1:0.5

All_Parameters=[All_Parameters; fe];

All_Parameters=[All_Parameters; f];

All_Parameters=[All_Parameters; RR];

All_Parameters=[All_Parameters; rho];

psy = 1.09270833; % half chisel edge angle (125°)

kab = 51.6; % N/mm^2 or MPa3

nc = 0.19233; % chip flow angle (11°)

Y = diff(y);

l1 = int(sqrt(1+Y^2),pl,r);

l = l1;

dl = diff(l,r); % Cutting edge model

for t=0.1:0.05:0.2

All_Parameters=[All_Parameters; t];

cl = t/(sin(3.147-psy)); % chisel edge dia

pl = t*tan(((90-(180-125))*3.147)/180)

All_Parameters=[All_Parameters; pl];

for r = RR

w = asin(t/r); % intermidiate angle

E = atan(tan(w)*cos(rho)); % intermidiate Angel

i = asin(sin(w)*sin(rho)); % inclination Angle

for d = 25:5:30

d0 = d*3.147/180

All_Parameters=[All_Parameters; d0];

delta = ((2*r)/(2*r))*tan(d0); % helix angle at any point

gr = (tan(delta)*cos(w))/(sin(rho)-(cos(rho)*tan(delta)*sin(w)));

% intermidiate angle

gn = gr - E; % normal rake

G = gn * 180/3.147

All_Parameters=[All_Parameters; G];

Ln = (19.1 + (0.29.*gn.*(180/3.1447))).*(3.147/180);

LN = Ln * 180/3.147 % friction angle

All_Parameters=[All_Parameters; LN];

pn = (atan(1./(1-tan(gn)))-Ln);

Pn = pn *180/3.147 % normal shear angll

t1 = (f.*sin(rho).*cos(E))./2; % depth of cut

All_Parameters=[All_Parameters; Pn];

tn = atan(tan(Ln).*cos(nc)-gn);

Tn = tn *180/3.147

All_Parameters=[All_Parameters; Tn];

%Forces

dfs = (kab.*t1.*dl)./(sin(tn));

% elements of force components

dF1c = (dfs .*cos(pn-gn))./cos(tn);

dF1t = dfs .* sin(pn-gn)./cos(tn);

dF1r = ((dF1c.^2 + dF1t.^2).^0.5).*sin(Ln).*tan(nc);

% force acting on elements

dFc = dF1c.*cos(i) + dF1r.*sin(i);

dFt = dF1t;

dFr = dF1r.*cos(i)-dF1c*sin(i);

A1 = (sin(Ln-gn-E).*sin(rho))-(tan(nc).*sin(Ln).*cos(rho)); % Constants

A2 = (cos(i).*cos(pn-gn)) - (sin(i).*sin(Ln).*tan(nc)); % Constants

syms x

X = r

All_Parameters=[All_Parameters; X];

dFl = int((kab.*f.*cos(E).*A1.*x)./(2.*sin(pn).*cos(tn).*(sqrt(x^2-t^2))),cl,X);

dCl = int((kab.*f.*cos(E).*A2.*x^2)./(2.*sin(pn).*cos(tn).*(sqrt(x^2-t^2))),cl,X);

Fl = double(dFl) % Thrust Force

All_Parameters=[All_Parameters; Fl];

Cl = double(dCl) % Torque

All_Parameters=[All_Parameters; Cl];

R = r;

F = dFl ;%+ Fc21; %Total force

Ft = double(F)

All_Parameters=[All_Parameters; Ft];

C = dCl ;

Ct = double(C)

All_Parameters=[All_Parameters; Ct];

end

end

disp(All_Parameters)

end

Matrix = [Matrix, All_Parameters]; %for contacting columns horizontally

end

T = array2table(Matrix);%,'VariableNames',column_names);

writetable(T,excelsheet_name)
